# Molecular Recognition of PTS-1 Cargo Proteins by Pex5p: Implications for Protein Mistargeting in Primary Hyperoxaluria

**DOI:** 10.3390/biom5010121

**Published:** 2015-02-13

**Authors:** Noel Mesa-Torres, Nenad Tomic, Armando Albert, Eduardo Salido, Angel L. Pey

**Affiliations:** 1 Department of Physical Chemistry, Faculty of Sciences, University of Granada, Av. Fuentenueva s/n, 18071 Granada, Spain; E-Mail: noelmesa@ugr.es; 2 Center for Biomedical Research on Rare Diseases (CIBERER), University Hospital of the Canary Islands and CIBICAN, University of La Laguna, 38320 Tenerife, Spain; E-Mails: ntomic@ull.es (N.T.); esalido@ull.es (E.S.); 3 Departamento de Cristalografía y Biología Estructural, Instituto de Química-Física “Rocasolano”, Consejo Superior de Investigaciones Científicas, C/Serrano 119, 28006 Madrid, Spain; E-Mail: xalbert@iqfr.csic.es

**Keywords:** peroxisomes, protein import, primary hyperoxaluria type I, peroxin 5, protein:protein interactions, binding energetics, structure-energetic correlations

## Abstract

Peroxisomal biogenesis and function critically depends on the import of cytosolic proteins carrying a PTS1 sequence into this organelle upon interaction with the peroxin Pex5p. Recent structural studies have provided important insights into the molecular recognition of cargo proteins by Pex5p. Peroxisomal import is a key feature in the pathogenesis of primary hyperoxaluria type 1 (PH1), where alanine:glyoxylate aminotransferase (AGT) undergoes mitochondrial mistargeting in about a third of patients. Here, we study the molecular recognition of PTS1 cargo proteins by Pex5p using oligopeptides and AGT variants bearing different natural PTS1 sequences, and employing an array of biophysical, computational and cell biology techniques. Changes in affinity for Pex5p (spanning over 3–4 orders of magnitude) reflect different thermodynamic signatures, but overall bury similar amounts of molecular surface. Structure/energetic analyses provide information on the contribution of ancillary regions and the conformational changes induced in Pex5p and the PTS1 cargo upon complex formation. Pex5p stability *in vitro* is enhanced upon cargo binding according to their binding affinities. Moreover, we provide evidence that the rational modulation of the AGT: Pex5p binding affinity might be useful tools to investigate mistargeting and misfolding in PH1 by pulling the folding equilibria towards the native and peroxisomal import competent state.

## 1. Introduction

Peroxisomes are highly specialized organelles ubiquitously found in eukaryotic cells and involved in specific metabolic processes, such as fatty acid β-oxidation and glyoxylate detoxification [1,2]. Proper peroxisomal biogenesis relies on the import of proteins synthesized in the cytosol by two peroxisomal targeting sequence (PTS) pathways [3]. The PTS type 1 (PTS1) pathway is the most widely used, and depends on the interaction of the peroxin 5 (Pex5p) receptor with a C-terminal peptide with a (S/A/C)-(K/R/H)-(L/M) consensus sequence [1]. The PTS type 2 (PTS2) pathway uses a degenerated N-terminal nonapeptide that is cleaved upon import, and seems to interact with a Pex5p/Pex7p complex in mammals [3]. The structural basis of the recognition of PTS1 cargo proteins has been the focus of a few remarkable studies, which have shown that the interaction of different cargo proteins with Pex5p may involve different structural mechanisms [4,5,6], and therefore the thermodynamic fingerprint for this recognition may diverge among cargo proteins. Indeed, differences in the molecular recognition patterns and therefore the affinity of Pex5p for their cargo proteins may correlate with their expression levels, thus allowing the existence of a pool of Pex5p-cargo protein complex levels compatible with proper peroxisomal biogenesis [7]. However, detailed structure/energetic analyses for the molecular recognition of cargo proteins by Pex5p is lacking.

Human alanine: glyoxylate aminotransferase (AGT) is a PLP-dependent enzyme that catalyzes the conversion of alanine to pyruvate and glyoxylate to glycine in hepatocytes, and its main role is the detoxification of glyoxylate in peroxisomes, which is its normal localization in humans [2,8,9]. Genetic mutations in the *AGXT* gene impairing AGT function are the cause of primary hyperoxaluria type I (PH1), a rare genetic disease inherited in an autosomal recessive manner [2,9,10]. Mutations often decrease the ability of AGT to fold into active dimers and in some cases also lead to mitochondrial mistargeting of the enzyme [11,12,13]. Misfolding and mistargeting in PH1 seems to be primarily caused by protein stability problems [9,12,14], which can be overcome by supplementation with vitamin B6 thus boosting intracellular levels of PLP and AGT stability [12,15]. The presence of two polymorphisms (p.P11L and p.I340M) that constitute the minor allele predispose the AGT enzyme towards deleterious mutations by reducing AGT intracellular foldability, establishing a folding and stability threshold separating health and disease [16]. The most common mutation in the minor haplotype, p.G170R (p.P11L/p.G170R/p.I340M), named AGT-LRM for short, is also the best characterized mistargeting AGT variant. Remarkably, the fate of a given PH1 causing variant (e.g., misfolding/aggregation in peroxisomes *vs.* mitochondrial mistargeting) depends on the expression conditions used [11,12,13], thus suggesting that the complex kinetic and thermodynamic relationships altered by the mutation and controlling AGT folding, misfolding and mistargeting inside cells can be modulated to a certain extent, and thus, could be exploited to provide new pharmacological treatments for this disease.

In the present study, we investigate the thermodynamic and structural basis of the molecular recognition of cargo proteins by Pex5p employing different natural PTS1 sequences with widely different affinities, using peptides and the corresponding sequences introduced at the PTS1 sequence of the human AGT. We hypothesize that the subcellular localization of human AGT variants is the result of a balance between interactions of the folded AGT dimer with the peroxisomal import machinery and binding of a partly folded AGT to cellular chaperones that facilitate mitochondrial import. Thus, we have analyzed Pex5p binding to the wild-type AGT protein and the mistargeting AGT (AGT-LRM) displaying different C-terminal PTS1 sequences. Our results suggest that beyond the specific interactions of Pex5p with the PTS1 sequence, additional ancillary regions in the cargo protein as well as conformational and dynamic changes in both cargo and receptor proteins may contribute significantly to the binding affinity and therefore display different thermodynamic signatures. Moreover, these designed AGT variants can be used to tune the intracellular misfolding and mistargeting of PH1-causing variants, and may be useful tools to get further insight into the molecular mechanisms underlying PH1.

## 2. Results and Discussion

### 2.1. Modeling the Interaction of AGT and Pex5p-pbd

**Figure 1:**
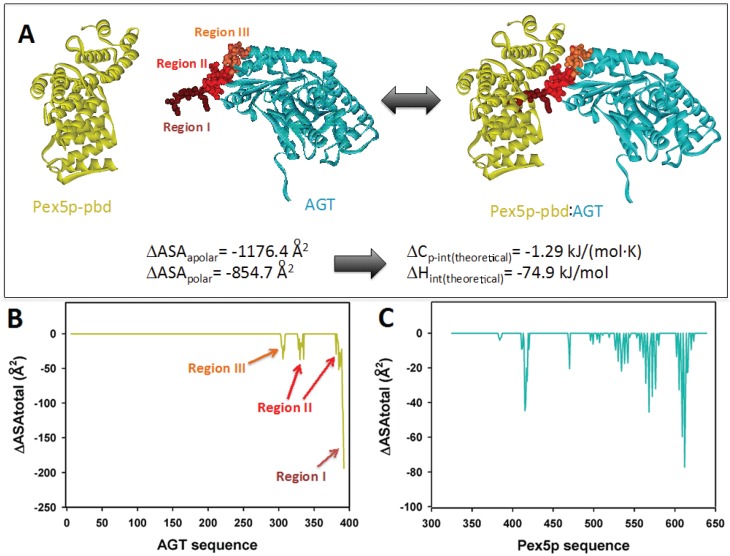
(**A**) Molecular architecture of the AGT:Pex5-pbd complex (PDB code: 3R9A), indicating the three regions in AGT involved in the interaction surface. The amount of polar and apolar surface buried is determined from this complex and used to estimate theoretical values of intrinsic enthalpy and heat capacity of binding using Equations (1) and (2) (**B**) and (**C**) Changes in accessible surface at the residue level for AGT (**B**) and Pex5p-pbd (**C**) upon complex formation.

The interaction between AGT and the PTS1 binding domain of Pex5p (Pex5p-pbd) exclusively involves the C-terminal domain of AGT (residues 283–392) (PDB: 3R9A; Figure 1A). While the overall conformation and activity of AGT are not significantly affected by the interaction, a conformational change is observed in the C-terminal bundle domain of Pex5p-pbd [5]. The reported structure shows three topologically distinct regions of AGT interacting in the complex (Figure 1A): (i) residues 389–392, which constitute the *minimal* PTS1 sequence and interact with the central cavity of the TPR domains of Pex5p-pbd. This region forms an interface with the receptor of about 600 Å^2^ [5]; (ii) residues 381–388 (helix α13) and 327–330 (contained in the *ancillary targeting sequence* or PTS1A; [17]), and recently named the *extended PTS1* [5]. Mutation of residues in this region show mild to moderate changes in affinity (about 2-fold lower affinity in Y330W and A328W, and about 5-fold in Y330A; [5]); (iii) residues 303–307. Regions II and III form an additional surface of about 400 Å^2^ [5]. Changes in molecular surface upon complex formation at the residue level are shown in Figure 1B,C.

Previous work with model peptides has shown that the recognition of PTS1 peptides by Pex5p-pbd resides to a large extent in the C-terminal tripeptide (positions −1 to −3; [18]), even though residues upstream of this tripeptide may also contribute to the binding affinity by at least two orders of magnitude [19]. However, these studies focused on the binding affinity (*i.e.*, binding free energy, Δ*G*, which is related to the association constant *K*_a_ by Δ*G* = −R·T·ln*K*_a_), and did not investigate the possible effect on the enthalpic (Δ*H*) and entropic (−*T*Δ*S*) contributions (Δ*G* = Δ*H* − *T*Δ*S*), which may suggest a link between changes in binding affinity with structural and dynamic differences in the complexes [20,21,22]. Very recently, Fodor *et al.* [6] characterized the impact of specific residues of the human AGT PTS1 octapeptide sequence on the affinity for Pex5p-pbd by alanine scanning mutagenesis and isothermal titration calorimetry using full-length human AGT. Mutations at residues Q385, P388 and K389 (positions −8, −5 and −4 in AGT, respectively) had little effect on binding affinity (lower than 2-fold). Interestingly, the K389A mutant showed only 1.8-fold lower affinity but reduced the enthalpic penalization to binding by 10 kJ/mol. These findings are difficult to be solely explained by changes in molecular buried surface and alternatively may reflect significant structural reorganization in the complex and large enthalpy/entropy compensations. Mutations at the −3 and −2 positions have a much larger effect on binding affinity. The K390A *back-to-consensus* mutation (-KKL to -AKL) increases the binding affinity for the Pex5p-pbd receptor, and this effect is mainly explained by a 4 kJ/mol decrease in the enthalpic penalization, likely due to the shrinkage of the binding site and optimization of short-range interactions reported by X-ray crystallography [6]. Conversely, the K391A variant (that forms the very weak -KAL tripeptide) showed no detectable binding, which is in turn compensated in the double mutant K390A/K391A apparently due to structural optimization of specific interactions in the binding sites (further supported by the decrease in enthalpic penalization of 6 kJ/mol). The results from this elegant study suggest that the recognition of PTS1 cargo proteins by Pex5p is highly plastic from both structural and energetic viewpoints.

**Figure 2:**
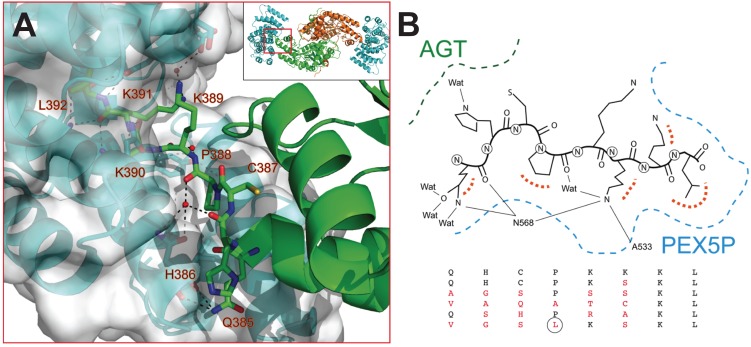
(**A**) Structural details of the interaction between AGT and Pex5p-pbd (PDB: 3R9A) (**B**) Overview of the modeled structures of AGT with modified PTS1 sequences.

The availability of crystal structures for AGT: Pex5p-pbd complexes allow the estimation of some thermodynamic theoretical values corresponding to the intrinsic binding process (excluding conformational changes and ionization effects coupled to binding). Upon complex formation, total changes in polar and apolar surface (ΔASA_polar_ and ΔASA_apolar_) are 855 Å^2^ and 1176 Å^2^ (Figure 1A), therefore yielding theoretical values for the intrinsic Δ*C*_p_ and Δ*H* values upon binding of −1.29 kJ/(mol·K) and −74.9 kJ/mol based on Equations (1) and (2) (see also [20]). These analyses set reference values to be contrasted with the calorimetric analyses performed in the present work, and to infer thermodynamic properties due to conformational changes. In the case that we use the complex AGT-WT-Pex5p-pbd to model different PTS1 sequences (Table 1 and Figure 2), we will obviously obtain a constant contribution from region III, and likely a variable contribution from regions I and II in the AGT (Figure 1A and Figure 2). Similarly, conformational changes in the C-terminal bundle of Pex5p-pbd are expected to remain constant across different modeled PTS1 sequences unless binding of AGT variants causes different long-range effects, while some differences in the TPR cavity are expected. To determine these intrinsic theoretical values for the different PTS1, we have modeled them using the available crystal structures, and the calculations of ΔASA_polar_ and ΔASA_apolar_ for the different PTS1 were carried out using the entire AGT protein and also for the 385–392 C-terminal octapeptide (compiled in Table 2). It must be noted that the overall conformations of the three AGT-Pex5p-pbd crystallographic complexes available are very similar, and they mainly differ in the adaptation of Pex5p-pbd cavity to accommodate the reduced size of the mutated residues at positions −3 and −2 [5,6]. Thus, it is likely that the analyses of the pattern of interactions between AGT and Pex5p-pbd (Figure 2B) can be used to explain our subsequent calorimetric analyses on the five AGT mutated versions to some extent. The C-terminal residues of AGT protrude into a central cavity of Pex5p-pbd forming both hydrogen bonds and hydrophobic interactions. Of them, residues K390, K391 and L392 are partially buried in a Pex5p-pbd central cavity while residues from Q385 to K389 are placed at the interface between AGT and the receptor. This suggests that mutations in the far C-terminal residues affect the structure of the receptor, while mutations in the rest of the tail may alter the relative position of the receptor with respect to AGT. To provide insights to this idea, we modeled the sequence of the five mutant proteins using as a template the structure of the AGT-Pex5p-pbd K390A mutant complex. Our models show that most mutations could be accommodated in the complex with minimal modifications of the original structure as none of them is totally buried in the complex interface. Conversely, the large and hydrophobic Leu side chain at 388 would require a structural reorganization (Figure 2B).

**Table 1:** PTS1 octapeptide sequences studied in this work. The protein sequence in which these octapeptides are naturally found, as well as their previously determined dissociation constant, are indicated [7]. In red, we indicate the variations in sequence found when the peptides are compared with the PTS-1 sequence of human AGT. N.Det.: not determined.

PTS1 Sequence	Enzyme	Sequence	*K*_d_ (nM)
AGT	AGT	QHCPKKKL	13490
AGT-SKL	AGT + consensus tripeptide	QHCPKSKL	N.Det.
BFE	L-Bifunctional enzyme	AGSPSSKL	1096
HMG	3-Hydroxy-3-methylglutaryl-CoA lyase	VAQATCKL	1995
CRA	Carnitine acyl-transferase 1	QSHPRAKL	59
ACO	Acyl-CoA oxidase 3	VGSLKSKL	1.6

**Table 2:** Changes in accessible surface (ΔASA) predicted for the AGT-Pex5p-pbd complexes studied here. Data correspond to the full length AGT with different PTS1 sequences.

PTS1 Sequence	ΔASA (for AGT) (Å^2^)		ΔASA (for Peptide) (Å^2^)	
	Apolar	Polar	Apolar	Polar
AGT	−1176	−855	−802	−579
AGT-SKL	−1075	−812	−718	−599
BFE	−1069	−659	−673	−450
HMG	−1100	−703	−715	−493
CRA	−1126	−776	−731	−580
ACO	−1168	−713	−783	−519

### 2.2. PTS1 Sequence Dependence of the Interaction between AGT and Pex5p-pbd: Enthalpy-Entropy Compensations

**Figure 3:**
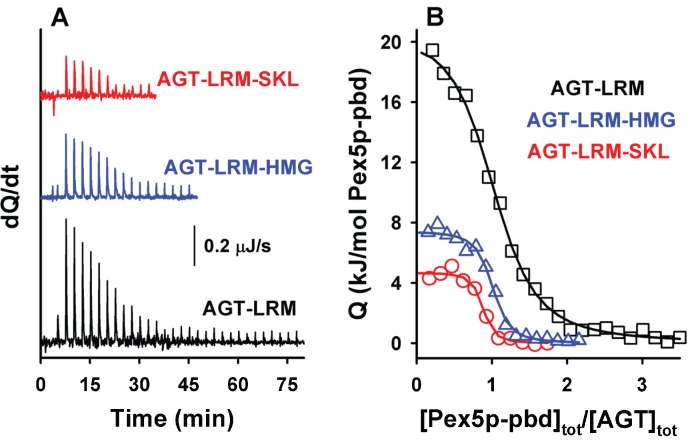
ITC binding analysis of AGT-LRM and AGT-LRM-SKL proteins with Pex5p-pbd. Thermograms (**A**) and binding isotherms (**B**) at 25 °C for AGT-LRM, AGT-LRM-HMG and AGT-LRM-SKL; AGT-LRM (15 μM in subunit) was titrated using Pex5p-pbd 350 μM (29 injections of 1.2 μL) while AGT-LRM-HMG and AGT-LRM-SKL (20 μM in subunit) was titrated using Pex5p-pbd 250 μM (12–16 injections of 2–2.2 μL).

Binding of AGT to Pex5p-pbd show a stoichiometry of 1:1 (Figure 3 and Table 3, see also [5,12]). Interestingly, the wild-type (AGT) and disease-causing AGT-LRM mutant show virtually identical affinities as well as thermodynamic signatures (Table 3). Introduction of the consensus -SKL tripeptide into either AGT or AGT-LRM variants produces a 50-fold increase in the affinity for the receptor, as well as a decrease in the enthalpic penalty of 16 kJ/mol. A similar behavior has been recently described for the -AKL tripeptide (mutation K390A of AGT; [6]), and accordingly, may also reflect an optimization of the interactions between the PTS1 sequence of AGT and the Pex5-pbd in the consensus -SKL PTS1 sequence.

**Table 3:** Thermodynamic binding parameters for the interaction of Pex5p-pbd with AGT and AGT-LRM variants at pH 7.4 and 25 °C. Data are mean ± s.d. of three independent titrations.

AGT Variant	N	K_d_	ΔG (kJ/mol)	ΔH (kJ/mol)	-TΔS (kJ/mol)	ΔC_p_^a^ (kJ/mol·K)
AGT	1.0 ± 0.1	1.4 ± 0.2 μM	−33.5 ± 0.4	21.7 ± 3.7	−55.2 ± 1.2	−2.5 ± 0.4
AGT-LRM	1.1 ± 0.1	1.6 ± 0.4 μM	−33.0 ± 0.8	21.3 ± 0.4	−55.3 ± 3.6	−2.7 ± 0.2
AGT-SKL	0.8 ± 0.1	44 ± 10 nM	−43.1 ± 0.9	6.3 ± 0.8	−49.4 ± 1.7	−2.7 ± 0.1
AGT-LRM-SKL	0.9 ± 0.1	65 ± 27 nM	−41.0 ± 0.7	5.0 ± 0.3	−46.0 ± 0.5	−2.6 ± 0.1

^a^ ΔC_p_ values are determined from the linear dependence of ΔH on temperature.

**Figure 4:**
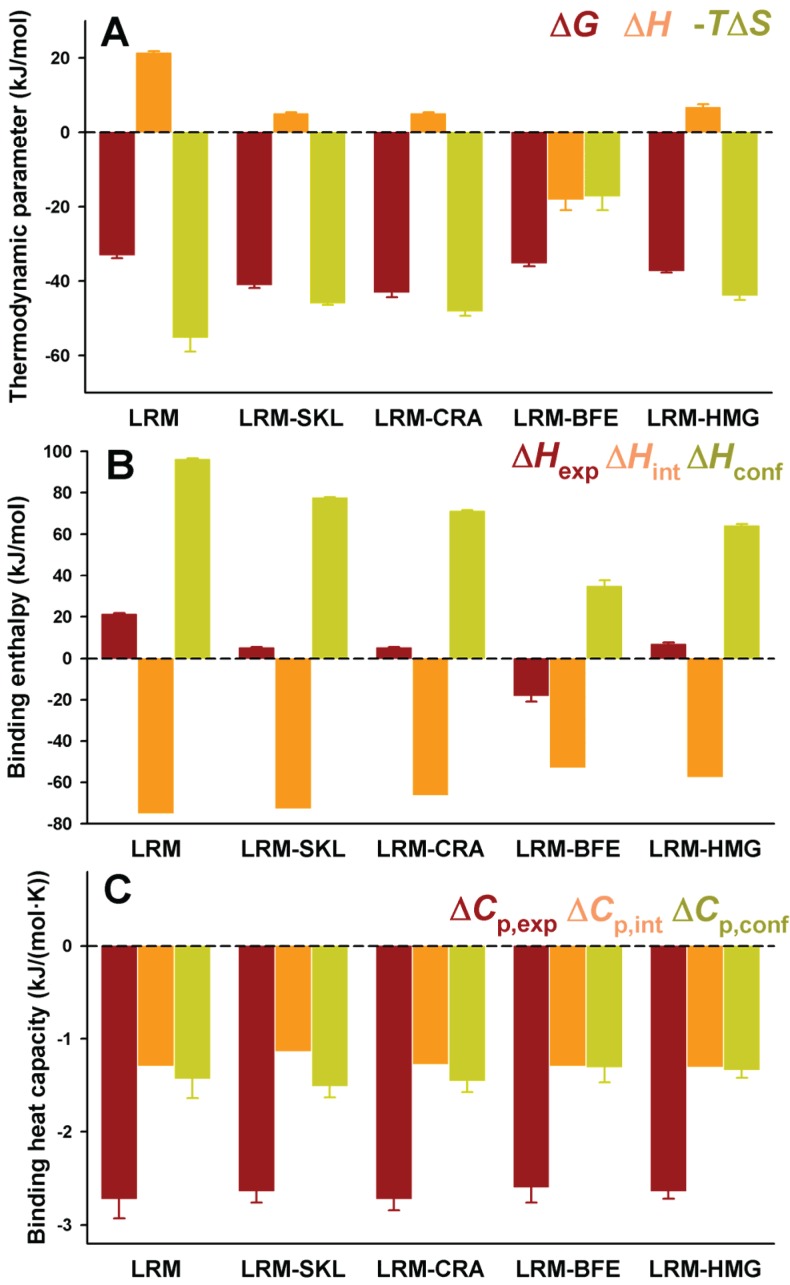
Thermodynamic binding properties for the interaction of protein variants on the AGT-LRM background with Pex5p-pbd. (**A**) Free energy (Δ*G*), enthalpy (Δ*H*) and entropy (−*T*Δ*S*) contributions to the binding reaction; (**B**,**C**) Thermodynamic dissection of binding enthalpies (**B**) and heat capacity changes (Δ*C*_p_, **C**) into their intrinsic contribution (estimated from polar and apolar ΔASA using the crystal structure for the complex; Figure 1) and the contribution from conformational changes. In (**A,B**), data are mean ± s.d. for three independent experiments at 25 °C.

As noted above, residues upstream from the C-terminal tripeptide of the PTS1 sequence may strongly modulate binding affinity [18,19]. Therefore, we have introduced PTS1 octapeptides previously described by [7] and displaying widely different binding affinities for Pex5p-pbd (Table 1). As expected from this previous report, we observed an increase in binding affinity compared to the natural PTS1 sequence of AGT (Figure 4A and Table 4). On the AGT-LRM background, several PTS1 sequences (AGT-SKL, CRA and HMG, Table 1) increased the affinity with a significant reduction of the enthalpic penalties (about 16 kJ/mol), while the BFE sequence (showing only a 2-fold increase in affinity) shows much favorable enthalpic contribution (about 35 kJ/mol) and much less favorable entropic contribution to binding (Figure 4A). These changes in the enthalpic contributions to binding could be at least partly explained by structural optimization of the PTS1 sequence at the Pex5p-pbd binding site similar to that proposed for the AGT–SKL variant (Table 3) and found for the K390A AGT mutant [6]. We must also note that the changes in binding affinity found are relatively modest in most of the cases due to significant enthalpy/entropy compensations (Figure 4A and [6]), highlighting the complex sequence/energetic dependence of the molecular recognition of PTS1 cargo proteins by Pex5p-pbd, which goes beyond the –SKL consensus tripeptide.

**Table 4:** Thermodynamic binding parameters for the interaction of PTS1 peptides and AGT-LRM variants with Pex5p-pbd at pH 7.4 and 25 °C determined by ITC. N.Det. not determined.

AGT ^a^	*K*_d_	Peptide ^b^	*K*_d_
AGT-LRM	1.6 ± 0.4 μM	pAGT	19 μM
AGT-LRM-SKL	65 ± 27 nM	pAGT-SKL	29 nM
AGT-LRM-CRA	40 ± 25 nM	pCRA	157 nM
AGT-LRM-BFE	0.74 ± 0.25 μM	pBFE	145 nM
AGT-LRM-HMG	0.28 ± 0.04 μM	pHMG	N.Det.
AGT-LRM-ACO	N.Det.	pACO	14 nM

^a^ Data are mean ± s.d. of three independent titrations; ^b^ Data are means of two independent titrations.

The experimental enthalpic contributions to binding may be structurally rationalized using well-known structure/energetic relationships [20]. Since the binding enthalpies are not dependent on the chosen buffer (giving similar values in HEPES and phosphate; data not shown [23]), contributions from ionization events upon binding should be ruled out. Therefore, the experimental binding enthalpies can be dissected in two main terms: (i) Δ*H*_int_, arising from the surface area (polar and apolar) buried upon binding; this term can be calculated from the crystal structures (Figure 1) and models provided in this work (Figure 2 and Table 2); (ii) Δ*H*_conf_, which arises from conformational changes in the cargo protein and Pex5p-pbd receptor upon binding. This term is evaluated from the difference between the experimental and intrinsic binding enthalpies. We must note that this energetic dissection is aimed to detect large differences between experimental and structure-derived theoretical values, thus allowing propose the existence of different conformational changes upon cargo binding, but not provide a detailed structural-energetic description of complex formation that could be a difficult task even if high-resolution crystal structures would be available for the complexes. These two terms (Δ*H*_int_ and Δ*H*_conf_) have been estimated for the five variants on the AGT-LRM background experimentally studied (Figure 4B). Δ*H*_int_ seems to moderately vary among these five variants, from −57 kJ/mol to −75 kJ/mol (mean ± s.d. = −65 ± 10 kJ/mol), being always favorable to binding. However, Δ*H*_conf_ largely varies among these AGT proteins, with values ranging from 35 to 96 kJ/mol (mean ± s.d. = 69 ± 22 kJ/mol), and is thus always unfavorable to binding. We must finally note that the contributions from Δ*H*_conf_ and Δ*H*_int_ show little or no correlation with the binding affinity, supporting complex enthalpy/entropy compensations ultimately leading to the given binding affinity for Pex5p-pbd-AGT complex formation.

A similar dissection has been performed for the binding heat capacities, for which the experimental values are virtually identical for the five AGT-LRM variants studied (Figure 4C). In this case, both contributions are of the same sign and contribute similarly to the experimental value (mean ± s.d. of −1.26 ± 0.07 and −1.40 ± 0.08 kJ/mol, for the intrinsic and conformational components, respectively), and thus, very small changes in each contribution among the AGT proteins studied. This likely reflects the different contributions of polar and apolar surface burial to the binding enthalpies and heat capacities (see Equations (1) and (2)).

### 2.3. Interaction between PTS1 Nonapeptides and Pex5p-pbd Provide Insight into the Contribution from Ancillary Regions to Binding Energetics

**Figure 5:**
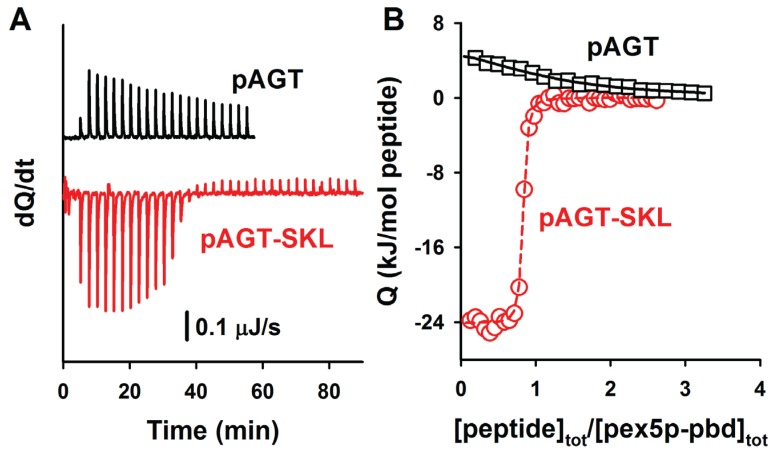
ITC analysis of the binding of the peptides pAGT and pAGT-SKL to Pex5p-pbd. Thermograms (**A**) and binding isotherms (**B**) at 25 °C for pAGT (squares) and pAGT-SKL (circles); Pex5p 20 μM was titrated using 0.5 mM of pAGT and pAGT-SKL peptides (25–30 injections of 1.2–1.5 μL).

**Figure 6:**
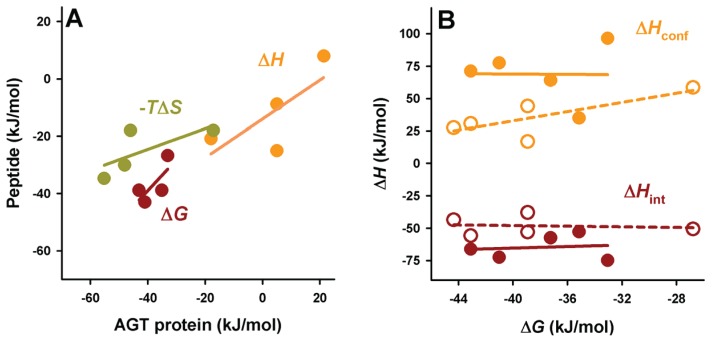
Context dependence of binding thermodynamics to Pex5p-pbd. (**A**) correlation of binding thermodynamic parameters between AGT proteins and PTS1 nonapeptides; (**B**) Binding free energies show little or no correlation with the different contributions (conformational or intrinsic) to binding enthalpies. In panel b, closed symbols are for AGT proteins and open symbols for PTS1 nonapeptides.

Representative ITC binding analyses of nonapeptides (containing PTS1 octapeptides *plus* a N-terminal tyrosine residue) to Pex5p-pbd are shown in Figure 5. A comparison of the binding affinities of these PTS1 nonapeptides with their corresponding full-length AGT counterparts reveals about one order of magnitude lower affinity for the peptides (Table 4; with the exception of AGT-SKL, that is close to the detection limit of ITC for a direct titration), in reasonable agreement with previous reports for AGT and SCP2 proteins [5,6,7]. The lower affinity in the peptides likely reflects the contributions from ancillary regions in the full-length cargo protein to complex formation, even though other minor contributions (such as electrostatic effects due to the presence of a charged N-terminal amine group in the peptides) should not be ruled out. Nevertheless, the difference in affinity between AGT proteins and PTS1 nonapeptides shows a reasonable correlation in terms of binding enthalpies and entropies (Figure 6A), suggesting the conservation of this context dependent cargo recognition (full-length protein *vs.* peptide) to some extent. This supports the idea that, overall, the binding mode of all AGT proteins to Pex5p-pbd is similar and mainly encoded in the C-terminal octapeptide sequence, while other ancillary regions in the AGT protein roughly add one order of magnitude to the binding affinity.

**Figure 7:**
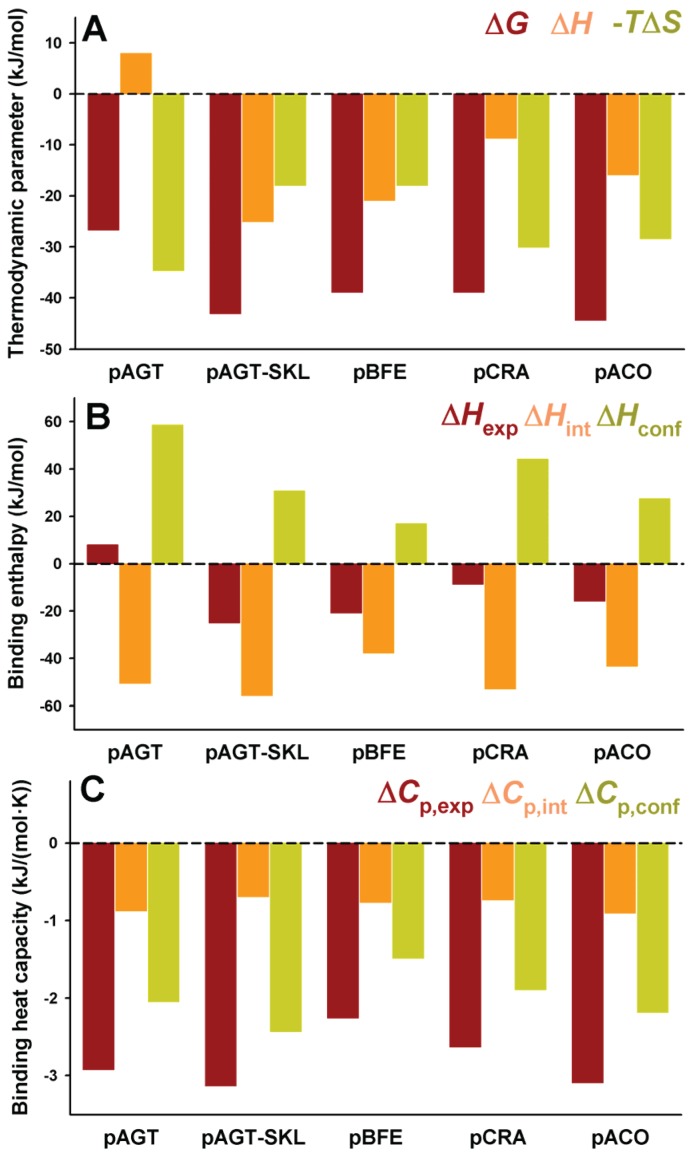
Thermodynamic binding properties for the interaction of PTS1 nonapeptides with Pex5p-pbd. (**A**) Free energy (Δ*G*), enthalpy (Δ*H*) and entropy (−*T*Δ*S*) contributions to the binding reaction; (**B**,**C**) Thermodynamic dissection of binding enthalpies (**B**) and heat capacity changes (Δ*C*_p_, **C**) into their intrinsic contribution (estimated from polar and apolar ΔASA using the crystal structure for the complex; Figure 1) and the contribution from conformational changes. In (**A,B**), data are means for two independent experiments at 25.

A comparative analyses of the intrinsic and conformational contributions to the experimental Δ*H* and Δ*C*_p_ value may provide some insight into this gain in affinity between full-length cargo protein and the nonapeptides (Figure 6 and Figure 7). In general, the peptides show a lower enthalpic penalization to binding, which indicates that a significant fraction of this penalization in the full-length protein arises from ancillary regions, and is somewhat entropically compensated. While the contributions to the enthalpy show similar nature in the full-length AGT and the peptides, the average favorable contribution from the intrinsic component is 17 kJ/mol lower in the peptide, while the conformational component reduces its penalization by about 33 kJ/mol, thus supporting the notion that the ancillary regions contribute favorably in terms of solvent exposure but unfavorably in terms of conformational changes (which is the main source of the enthalpic penalization to binding in the full-length AGT). As found for full-length proteins, the correlation between intrinsic and conformational components to the binding affinity is weak (Figure 6B). In the case of binding heat capacities, the behavior of the peptides is similar to that of the full-length protein, but as expected, the contribution of the intrinsic component is lower due to the smaller changes in solvent accessibility compared to the full-length protein.

### 2.4. In Vitro Stabilization of Pex5-pbd Upon Peptide Binding Correlate with Binding Affinity

**Figure 8:**
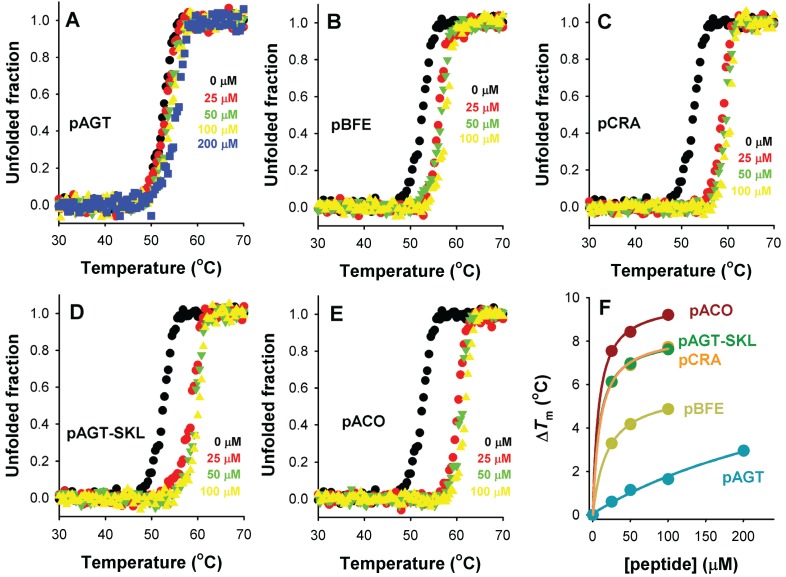
Thermal stabilization of Pex5p-pbd upon PTS1 nonapeptide binding. (**A**–**E**) show thermal denaturation profiles in the absence or presence of different peptide concentrations (as the colors indicated); (**F**) shows the thermal up-shift (Δ*T*_m_) of Pex5p-pbd as a function of peptide concentration.

**Figure 9:**
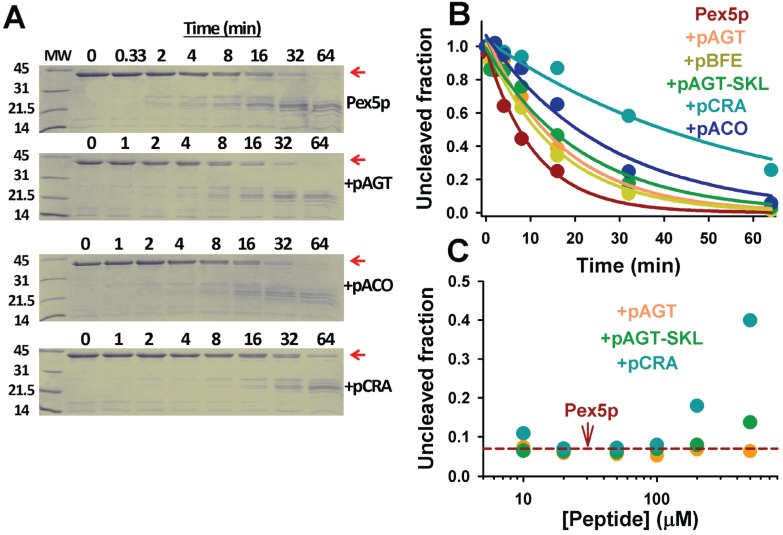
Proteolysis of Pex5p-pbd in the absence and presence of PTS1 nonapeptides. (**A**) SDS-PAGE gels of proteolysis kinetics of Pex5p-pbd in the absence or presence of peptides. MW indicates molecular weight markers in kDa, and the native band of Pex5p-pbd is indicated by a red arrow. Gels were stained with Coomassie blue brilliant; (**B**) Proteolysis kinetics of the native band of Pex5p-pbd. Lines are best-fits to a single exponential function; (**C**) Fraction of native Pex5p-pbd after 32 min proteolysis in the absence (horizontal dashed red line) and presence of different peptide concentrations. Thermolysin and Pex5p-pbd concentrations were 10 nM and 20 μM, respectively. In (**A**) and (**B**) peptide concentration was 500 μM.

Protein-protein interactions may lead to intracellular stabilization of the protein partners, and alterations in binding and subsequent stabilization may contribute to human disease [24,25]. Stabilization (at least *in vitro*) is often interpreted as the preferential binding of the partners as folded conformations rather than as non-native conformations, and in principle, the degree of stabilization may be related to binding affinity. We have thus tested whether binding of PTS1 sequences may stabilize Pex5p-pbd using our set of peptides, instead of AGT proteins because the latter show extremely high thermal and kinetic stabilities *in vitro* [12,26]. To this end, we have used two complementary approaches: thermal denaturation experiments monitored by circular dichroism (Figure 8), and proteolysis kinetics under native conditions (Figure 9).

Thermal denaturation of Pex5p-pbd is irreversible and kinetically controlled (data not shown [23]), yielding a single denaturation transition with a *T*_m_ of ~51 °C (Figure 8). Addition of increasing concentrations of PTS1 nonapeptides leads to a gradual stabilization of Pex5p-pbd (Figure 8A–E). Interestingly, the peptide concentration dependence of thermal stabilization correlates well with their corresponding binding affinities (Figure 8F), which is compatible with a scenario in which the Pex5p-pbd peptide complex is kinetically protected against thermal denaturation, and thus, that the amount of free Pex5p-pbd protein determines to a large extent the rate of protein denaturation. Therefore, we hypothesize that cargo binding to Pex5-pbd may stabilize both the receptor and the cargo protein intracellularly, and this stabilization is plausibly linked to the binding affinity and the levels of Pex5p-pbd and cargo proteins.

Proteolysis is a useful tool to examine the effect of ligand binding on protein stability and dynamics [27,28,29]. We have thus examined changes in Pex5-pbd dynamics upon peptide binding by determining the proteolysis kinetic pattern in the presence of thermolysin (Figure 9). Pex5p-pbd is very sensitive to proteolytic attack by thermolysin, and displays a half-life of about 8 min in the presence of a 1:2000 protease:protein ratio. Addition of PTS1 nonapeptides protect towards proteolysis without changing the partial proteolysis pattern (Figure 9A), from a mild 1.5-fold with p.AGT and p.BFE to a 5-fold in p.CRA (Figure 9B). This protective effect is concentration-dependent (Figure 9C) and correlates fairly well with their corresponding affinities, as also observed for thermal denaturation of Pex5-pbd. This suggests that proteolysis kinetics are reflecting peptide binding effects on Pex5p-pbd dynamics and/or conformational stability.

### 2.5. Partial Correction of the Mistargeting Phenotype in a Disease-Causing AGT Variant by Enhancing PTS1 Binding Affinity

Folding, misfolding and mistargeting of human AGT inside cells is a remarkably complex process [12]. Wild-type AGT is known to transiently interact with molecular chaperones (Hsp70 and Hsp90) on its way to reach the functional and peroxisomal competent dimeric state [26]. However, upon synthesis, PH1 disease-causing variants are known to interact more strongly with Hsp70, Hsp90 and Hsp60 chaperones [12,13,26,30], and this behavior is either observed for variants classically associated to mitochondrial mistargeting or to peroxisomal aggregation. Similarly, PH1 variants associated to mistargeting or aggregation are often thermally and kinetically unstable, especially in their apo-form [12,26]. In some cases, aggregation has also been linked to altered conformational dynamics of the N-terminal of human AGT [31]. On the basis of all these results, we have recently proposed that several steps of the intracellular folding and assembly of human AGT would be affected in PH1 variants, and thus, may be potentially targeted for pharmacological treatment of PH1. We must note that it is possible that mitochondrial mistargeting and/or misfolding leading aggregation may kinetically trap the human AGT protein along its folding/misfolding/mistargeting processes, and therefore simply targeting steps from an equilibrium perspective (for instance, using native-state ligands) may not be efficient as new therapeutic approaches. Nevertheless, the fact that a given PH1 variant may lead to either mitochondrial mistargeting or enhanced aggregation depending on the expression conditions in eukaryotic cells [11,12] suggests that human AGT is not kinetically trapped along its folding/misfolding/mistargeting pathways and its final fate is amenable to modulation by environmental conditions (temperature, the presence of osmolytes, ligands, and so on).

**Figure 10:**
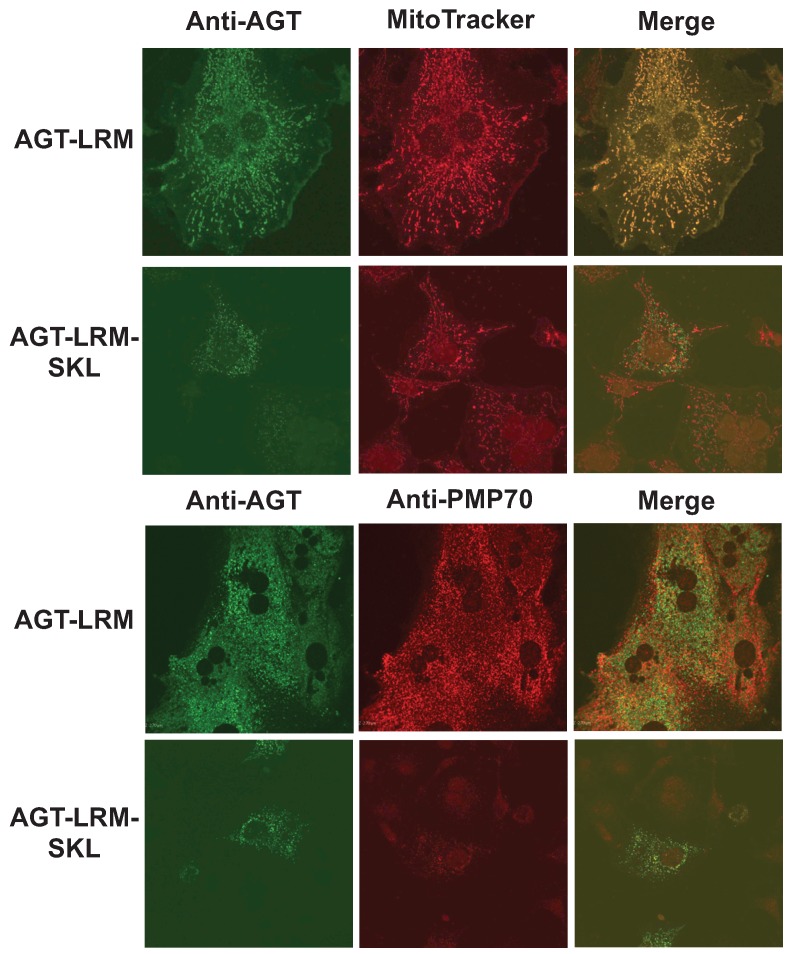
The consensus AGT-SKL sequence partially corrects mitochondrial mistargeting of AGT-LRM. Immunolocalization of AGT-LRM and AGT-LRM-SKL with mitochondrial (mitotracker) and peroxisomal (PMP-70) markers.

We have thus studied whether an increase in binding affinity of the human AGT native state for Pex5p could shift the equilibrium of human AGT along is folding/mistargeting pathways inside cells. To do so, we transiently transfected CHO cells with plasmids expressing the AGT-LRM variant, which is classically associated with mitochondrial mistargeting, and this variant with the PTS1 sequence mutated to the high affinity consensus –SKL C-terminal tripeptide (AGT-LRM-SKL). Confocal immunofluorescence images of a total of 443 cells were analyzed for the expression of AGT-LRM and AGT-LRM-SKL proteins and its colocalization with either mitochondria or peroxisomal markers is given in Table 5. As we show in Figure 10, the AGT-LRM variant is mostly found in mitochondria (R_r_ = 0.90, with 92% colocalization). However, in AGT-LRM-SKL, we observe a significant fraction of the protein in peroxisomes (84%), with a significant decrease in the colocalization with mitotracker in the mitochondria (R_r_ = 0.47, with 40% colocalization). The 95% confidence intervals for the percentage of mitochondrial localization in the two tested situations are disparate: in AGT-LRM 0.66 < m < 1.16 *vs.* AGT-LRM-SKL: 0.24 < m < 0.56. Similarly, the amount of AGT-LRM localized in peroxisomes (17%) appears significantly lower than AGT-LRM-SKL (84%). Both colocalization parameters correlate well with their respective Pearson’s coefficients: 0.39 in the AGT-LRM *vs.* 0.79 in AGT-LRM-SKL. The 95% confidence intervals are disparate in this case too: 0.15 < m < 0.44 *vs.* 0.68 < m < 1. However, the standard error for the study of AGT-LRM colocalization with peroxisomal marker PMP70 is relatively high, making the confidence interval wide.

**Table 5:** Descriptive statistics of intensity correlation coefficient-based (ICCB) parameters in CHO cells. Mean Pearson’s correlation coefficient (r) and the mean Manders’ percentage of colocalization (m) were determined as described previously [32], in the 4 experimental situations for: 1. AGT-LRM transfected cells in mitochondria; 2. AGT-LRM transfected cells in peroxisomes; 3. AGT-LRM-SKL transfected cells in mitochondria and 4. AGT-LRM-SKL transfected cells in peroxisomes. Abbreviations: AGT = alanine: glyoxylate aminotransferase, Mt = mitochondria, Px = peroxisomes. As a % of colocalization, we used Manders’ overlap R in situations 1 and 4, and split Manders’ M_1_ in situations 2 and 3.

	Experimental situations							
ICCB Parameters	1. AGT-LRM Mt: AGT + Mitotracker		2 AGT-LRM Px:AGT + PMP70		3. AGT-SKL Mt:AGT + mitotracker		4. AGT-SKL Px:AGT + PMP70	
	Pearson Rr	% Coloc	Pearson Rr	% Coloc	Pearson Rr	% Coloc	Pearson Rr	% Coloc
# cells	75		50		169		149	
r/m ± S.E.	0.90 ± 0.12	0.92 ± 0.12	0.39 ± 0.15	0.17 ± 0.15	0.47 ± 0.08	0.40 ± 0.08	0.79 ± 0.08	0.84 ± 0.08

This interpretation is also coherent with recent findings [33] that blocking mitochondrial import machinery partly resolves the mistargeting of AGT-LRM. Thus, the final localization of AGT-LRM seems to depend on the balance between the opposing forces of mitochondrial and peroxisomal import machineries, mediated by interactions with cellular chaperones and Pex5p, respectively.

## 3. Experimental Section

### 3.1. Peptides and Proteins

The peptides were synthesized by EZ Biolabs (Carmel, IN, USA) at >95% purity. The octapeptide sequences correspond to the C-terminal sequences of human peroxisomal proteins [7] that are compiled in Table 1. All peptides contained an extra N-terminal tyrosine residue to measure their concentration spectrophotometrically (ε_274nm_ = 1405 M^−1^·cm^−1^), and are referred to as PTS1 nonapeptides along the manuscript.

The PTS1 binding domain of human Pex5p (Pex5p-pbd; amino acids 235–602) and WT and LRM hAGT variants containing N-terminal hexa-histidine tags were expressed and purified as recently described [12]. Details on the protein constructs used can be found in the supplementary information. Mutations in the PTS1 sequence (Table 1) of AGT or AGT-LRM proteins were introduced by site-directed mutagenesis and the mutated sequences were confirmed by sequencing the entire AGT cDNA. Protein concentration was measured spectrophotometrically using ε_280nm_ = 50,880 M^−1^·cm^−1^ (Pex5p-pbd) or 46,820 M^−1^·cm^−1^ (AGT proteins in monomer).

### 3.2. Isothermal Titration Calorimetry

Calorimetric measurements were carried out using a MicroCal ITC_200_ microcalorimeter (MicroCal, Malvern Instruments) with 205.9 μL cell volume. All the experiments were performed using using 20 mM Na-HEPES (or Na-Phosphate), 200 mM NaCl, pH 7.4. AGT proteins (12–40 μM in protein subunit in the cell) were titrated using Pex5p-pbd (240–400 μM), while peptides (500 μM) were used to titrate Pex5p-pbd (20 μM in the cell). Each titration was initiated by a 0.5 μL injection followed by 14–30 injections of 1.2–2.5 μL (spaced 150 s). Heats of dilution were determined experimentally from blank titrations. The association constants (*K*_a_), binding enthalpy changes (Δ*H*), as well as the binding stoichiometries (N), were obtained by non-linear regression analysis using a one-independent-type-of-sites binding model implemented in the Origin 7.0. Software (Origin Lab Co., Northampton, MA, USA). The dissociation constant (*K*_d_), binding Gibbs free energy (Δ*G*) and entropy (Δ*S*) changes were obtained from basic thermodynamic relationships (Δ*G =* Δ*H* − *T*Δ*S*). Δ*C*_p_ values were obtained from the linear dependence of Δ*H* on temperature in a 10–35 °C temperature range.

### 3.3. Thermal Denaturation Studies

Thermal denaturation of Pex5p-pbd was evaluated by measuring changes in the ellipticity at 222 nm in thermal scans (2 °C/min scan rate) in a 20–90 °C range. These experiments were performed in Na-Hepes 20 mM, 200 mM NaCl pH 7.4, using 5 μM Pex5p-pbd and 0–200 μM peptides, and carried out in a Jasco J-710 spectroscopolarimeter equipped with a Peltier element and using 1 mm pathlength cuvettes. Melting curves were normalized considering pre- and post-transition linear baselines, and the normalized curves presented as unfolded fraction of Pex5p-pbd *vs.* temperature. The *T*_m_ corresponds to the temperature at which the unfolded fraction is 0.5.

### 3.4. Proteolysis by Thermolysin

Proteolysis experiments were performed using 10 nM thermolysin from *Bacillus thermoproteolyticus rokko* (Sigma-Aldrich, St. Louis, MO, USA), 20 µM Pex5p-pbd, in the absence or presence of the corresponding nonapeptides (10–500 µM). Proteolysis was performed at 25 °C in Na-Hepes 20 mM, 200 mM NaCl, CaCl_2_ 10 mM pH 7.4. Aliquots were withdrawn at different times and proteolysis quenched by the addition of EDTA (20 mM final concentration). Samples were analyzed by SDS-PAGE electrophoresis (12% PAGE), stained with Coomassie blue brilliant and gels were densitometered by ImageJ (http://rsbweb.nih.gov/ij/).

### 3.5. Structural Modeling

The crystal structure of the complex between AGT K390A mutant in complex with Pex5p-pbd (PDB code 4KYO; [6]) was used as a template to model different PTS1 sequences (compiled in Table 1). The three dimensional models were obtained with COOT [34]. Their geometry was optimized to minimize clashes while maintaining the correct stereochemistry of the model using COOT. A final automatic idealization cycle was preformed using Phenix [35]. Ribbon figures were produced using Pymol [36].

### 3.6. Structure-Energetic Calculations

To calculate the theoretical values for Δ*H*_int(25oC)_ and Δ*C*_p_ using the structure, the ΔASA for polar and apolar surfaces in the complex and for each protein individually (in Å^2^) were determined using a home-built software (kindly provided by Prof. Jose Manuel Sanchez-Ruiz, University of Granada, Spain), and following Equations (1) and (2) [20]:

(1) $$\Delta C_{p(theoretical)}=-1.09\cdot \Delta ASA_{polar}+1.88\cdot \Delta ASA_{apolar}(in\frac{J}{mol\cdot K})$$

(2) $$\Delta H_{int(theoretical)}=-129.96\cdot \Delta ASA_{polar}-30.75\cdot \Delta ASA_{apolar}(in\frac{J}{mol})$$

### 3.7. Cell Cultures

Chinese hamster ovary (CHO) cells (ATTC, Rockville, MD USA) were grown in alpha-minimal essential medium (α-MEM, Lonza, Köln, Germany) supplemented with glutamine, penicillin/streptomycin and 5% fetal bovine serum. Cell transfections were performed using AGT cDNA variants (AGT-LRM or AGT-LRM-SKL) subcloned in pCIneo plasmids (Promega) with Transfast reagent (Promega, Madison, WI, USA), following manufacturer’s guidelines. After 24 h, cells were passed to plates containing 13 mm glass coverslips for immunofluorescence studies. Mitochondria in half the coverslips were labeled by incubation with Mitotracker (CMXRos, Invitrogen, Carlsbad, CA, USA) at the end of the culture. Cells were fixed with 2% paraformaldehyde 72 h after transfection and immunofluorescence was performed as previously described [12]. Images were acquired with a laser scanning confocal microscope (Olympus Inverted IX81, Tokyo, Japan) using a 60× objective with immersion oil.

For quantitative immune-colocalization, the system was optimized to detect two fluorescent signals in one specimen: one in the green channel detector for protein of interest AGT-LRM or AGT-LRM-SKL and the other in the red channel, for either mitochondria labeled with mitotracker or peroxisomes labeled with peroxisomal membrane protein 70 (PMP70) antibody. When the two were seen to overlap (colocalization), it was assumed to be due to fluorescently labeled molecules binding to very close or indeed identical spatial positions. Colocalization events (or the lack thereof) were analyzed quantitatively with FluoView software (Olympus, Tokyo, Japan) and ImageJ software (NIH, Bethesda, MD, USA) with the JACoP plugin, based on a global statistic approach that conducts an intensity correlation coefficient-based examination [32,37]. The Fisher transformation was applied to data obtained from ImageJ as well when calculating the mean values of colocalization coefficients and constructing the respective 95% confidence intervals. Descriptive statistical analysis of colocalization parameters (Pearson’s coefficient, Manders’ overlap coefficient and split Manders’ coefficients) was performed.

## 4. Conclusions

In this work, we have explored several aspects of the molecular recognition of cargo proteins by Pex5p not well investigated previously. By comparing the energetics underlying Pex5p binding to different PTS1 peptides and their counterparts in the full-length AGT cargo protein, we have provided structural and energetic insight into the contribution from different regions of the cargo protein to the binding affinity for Pex5p. Moreover, binding of cargo proteins lead to stabilization of the peroxisomal receptor, which may be exploited to modulate the intracellular fate of PTS1 cargo proteins. We also demonstrate this experimentally, in cultured cells, showing that mistargeting of the most common PH1 mutation can be corrected by rational modulation of its binding affinity for Pex5p, which may be a useful tool to find new therapeutic agents to treat PH1.
